# BMP4 preserves the developmental potential of mESCs through *Ube2s*- and *Chmp4b*-mediated chromosomal stability safeguarding

**DOI:** 10.1007/s13238-021-00896-x

**Published:** 2022-02-11

**Authors:** Mingzhu Wang, Kun Zhao, Meng Liu, Mengting Wang, Zhibin Qiao, Shanru Yi, Yonghua Jiang, Xiaochen Kou, Yanhong Zhao, Jiqing Yin, Tianming Li, Hong Wang, Cizhong Jiang, Shaorong Gao, Jiayu Chen

**Affiliations:** 1 Clinical and Translation Research Center of Shanghai First Maternity & Infant Hospital, Shanghai Key Laboratory of Signaling and Disease Research, School of Life Sciences and Technology, Tongji University, Shanghai, 200092 China; 2 Frontier Science Center for Stem Cell Research, Tongji University, Shanghai, 200092 China; 3 Key Laboratory of Spine and Spinal Cord Injury Repair and Regeneration of Ministry of Education, Orthopaedic Department of Tongji Hospital, Tongji University, Shanghai, 200065 China; 4 Institute for Regenerative Medicine, Shanghai East Hospital, School of Life Sciences and Technology, Tongji University, Shanghai, 200123 China; 5 Guangxi Key Laboratory of Genomic and Personalized Medicine, Nanning, 530021 China

**Keywords:** BMP4, pluripotency, chromosomal integrity, developmental potential, serum-free

## Abstract

Chemically defined medium is widely used for culturing mouse embryonic stem cells (mESCs), in which N2B27 works as a substitution for serum, and GSK3β and MEK inhibitors (2i) help to promote ground-state pluripotency. However, recent studies suggested that MEKi might cause irreversible defects that compromise the developmental potential of mESCs. Here, we demonstrated the deficient bone morphogenetic protein (BMP) signal in the chemically defined condition is one of the main causes for the impaired pluripotency. Mechanistically, activating the BMP signal pathway by BMP4 could safeguard the chromosomal integrity and proliferation capacity of mESCs through regulating downstream targets *Ube2s* and *Chmp4b*. More importantly, BMP4 promotes a distinct *in vivo* developmental potential and a long-term pluripotency preservation. Besides, the pluripotent improvements driven by BMP4 are superior to those by attenuating MEK suppression. Taken together, our study shows appropriate activation of BMP signal is essential for regulating functional pluripotency and reveals that BMP4 should be applied in the serum-free culture system.

**Supplementary Information:**

The online version contains supplementary material available at 10.1007/s13238-021-00896-x.

## Introduction

Mouse embryonic stem cells (mESCs) are derived from the inner cell mass (ICM) of the developing blastocyst and have the abilities of self-renewal and pluripotency to reconstitute embryonic lineages (Evans and Kaufman, 1981; Martin, 1981). It has been reported that culture conditions could greatly affect genome stability, transcriptome, epigenome, proliferation and differentiation capacity of mESCs (Ying et al., 2003; Habibi et al., 2013; Hassani et al., 2014; Kolodziejczyk et al., 2015; Ter Huurne et al., 2017; Lee et al., 2018). Traditionally, mESCs are cultured on a layer of mitotically inactivated fibroblasts called feeders, with fetal bovine serum (FBS) and leukemia inhibitory factor (LIF) (with the culture condition defined as the S condition and the mESCs as S-mESCs). FBS is considered important because it provides not only certain critical biological molecules such as albumin, apolipoproteins and biotin (Halliwell, 1988; Zheng et al., 2006; Uddin et al., 2020) but also growth-supporting factors such as bone morphogenetic protein 4 (BMP4), which stimulates downstream SMAD signaling pathways to activate inhibitor of differentiation (Id) genes (Baker et al., 1988; Ying et al., 2003). However, the composition of FBS is not fully determined and there might be undefined molecules engendering some conflicting signaling pathways. In addition, it has been demonstrated that mESCs cultured in the S condition are in a metastable status and exhibit volatile transcriptional and epigenetic profiles and, ultimately, functional heterogeneity among cells (Hayashi et al., 2008; Hackett and Surani, 2014).

Compared with the S condition with its battleground of competing signals, a chemically defined culture condition is considered to be better for pluripotency maintenance and easier to keep stem cells homogeneous. Specifically, a serum substitute, N2B27 (N2 and B27), combined with two small-molecule kinase inhibitors termed “2i” (PD0325901, a potent MEK1/2 inhibitor, and CHIR99021, a specific GSK3β inhibitor) can drive mESCs in a uniform transcriptional and epigenetic state, which is called ground state (Sato et al., 2004; Kunath et al., 2007; Ying et al., 2008). Thus, this highly chemically defined condition (N2B27 combined with 2i: 1 μmol/L MEKi, 3 μmol/L Gsk3βi and LIF, defined as N/2i condition and the cultured mESCs as N/2i-mESCs) is theoretically more reproducible than the conventional S condition. Specifically, N/2i condition does greatly improve the derivation and pluripotency maintenance of mESCs (Buehr et al., 2008; Ying et al., 2008; Czechanski et al., 2014). However, it was reported that this condition could lead to telomere defects such as shorter telomeres and chromosome fusion in mESCs, which further caused a defective adult development of all-ESC mice (Guo et al., 2018). As a comparison, these aberrations are not observed in the serum-based condition, indicating the serum-free condition still has certain shortcomings.

Proper growth factors are essential for mESCs to stabilize the pluripotent transcriptional network. The most representative one is LIF that is essential for both serum and serum-free condition. LIF helps to maintain mESCs in an undifferentiated state, through activating three major intracellular signaling pathways including the JAK/STAT3, PI3K/AKT and SHP2 /MAPK (He et al., 2006; Hirai et al., 2011). Another important growth factor is BMP4, which supports the self-renewal of mESCs by inhibiting mitogen-activated protein kinase (MAPK) pathways and thus this role was supposed to be replaced by MEKi (Ogawa et al., 2004; Ying et al., 2008). However, the commonly used MEKi-PD0325901, is a potent and strong inhibitor for suppressing the ERK1/2 cascade. And ERK1/2 deficiency would cause series of abnormalities including rapid telomere shortening, genomic instability and compromised self-renewal of mESCs such as reduced proliferation, G_1_ cell-cycle arrest and apoptosis (Chen et al., 2015a). In addition, it was recently reported that prolonged treatment with MEKi can result in widespread loss of DNA methylation and irreversible erasure of genomic imprints, which further impairs strict *in vivo* embryonic development and causes aneuploidy (Choi et al., 2017; Yagi et al., 2017). Notably, aneuploidy is a common problem in the establishment and culture of pluripotent stem cells, especially in induced pluripotent stem cells (iPSCs). Although aneuploidy does not greatly weaken cell proliferation, it does lead to severe developmental defects in mammals (Zhang et al., 2016). Besides, it might alert gene expression and induce tumorigenesis (Ben-David et al., 2014).

To solve these problems, certain modifications were made in this chemically defined condition. Specifically, an Src inhibitor CGP77675 was used to substitute for MEKi- PD0325901 (defined as N/a2i condition) (Shimizu et al., 2012) or the concentration of PD0325901 was reduced to 0.2 μmol/L (defined as N/t2i condition). Both of these modifications could largely avoid the erosion of imprints, sustain chromosomal integrity and promote the pluripotency of mESCs (Yagi et al., 2017). Interestingly, other differences between S- and N/2i-mESCs in terms of culture system components, including serum and chemically defined medium, were overlooked, either intentionally or unintentionally. For instance, whether N/2i lacks certain important factors contained in serum condition that might cause the reported defects needs to be further investigated. In addition, most of these studies did not include comparisons of multiple cell lines and lacked sufficient *in vivo* differentiation data (Yagi et al., 2017; Wu et al., 2020). Generally, chimera assay is widely used to assess the *in vivo* developmental potential of mESCs. However, classical chimerism was mainly determined by the artificial and qualitative assessment of the degree of chimeric agouti coat color, which lacks quantifiable standards and may lead to inaccurate conclusions. In addition, the germline transmission ability of mESCs was not fully investigated. Thus, an objective and convenient analysis system needs to be applied for a more accurate use in determining the developmental potential of mESCs.

In this study, we verified a superiority of serum over chemically defined condition by applying a quantitative fluorescence analysis of chimeric potency assay combined with RNA-seq analysis. We uncovered certain key signaling pathways that were deficient in widely used serum-free N/2i condition. Specifically, the impaired BMP signal in N/2i-mESCs dysregulates the expression of ubiquitin-conjugating enzyme E2S (*Ube2s*) and charged multivesicular body protein 4B (*Chmp4b*), both of which are required for correct chromosome segregation and precise regulation of cell cycle. Besides, many key genes and signal pathways critical for pluripotency were abnormally downregulated in N/2i-mESCs, which could be restored by BMP4, but not by N/a2i or N/t2i. Further analyses revealed the essential role of BMP4 in long term preservation of euploidy and *in vivo* developmental potential of mESCs, which works without altering phosphorylation of ERK1/2 or DNA methylation. More importantly, BMP4 exhibits a better pluripotency improvement compared to N/a2i or N/t2i which functions in optimizing MEK suppression. Taken together, our study reveals that BMP4 needs to be applied to maintain the chromosomal integrity and to remold *in vivo* differentiation potency of mESCs cultured in the serum-free system.

## Results

### N/2i-mESCs possess a restricted pluripotency compared to S/2i-mESC

**Figure 1:**
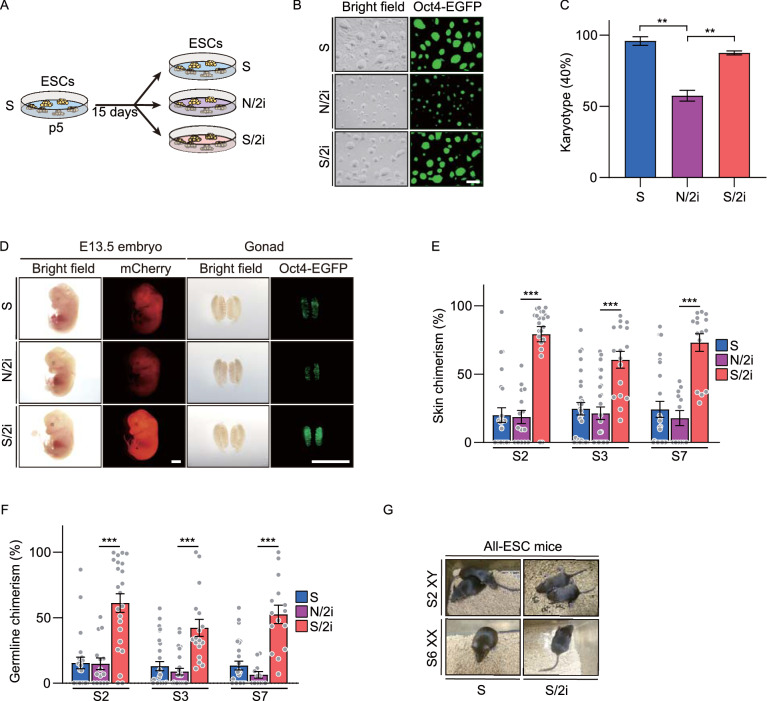
**S/2i-mESCs present a better developmental potential compared to N/2i-mESCs**. (A) Strategy for comparing the same mESC line cultured under different conditions. p, passage. (B) Representative morphological images of mESCs cultured under S, N/2i and S/2i conditions. The pictures were taken at 54 h after propagation of 10^5^ cells. Scale bar, 100 μm. (C) Karyotyping validation of S-, N/2i- and S/2i-mESCs. Note that N/2i-mESCs showed a high proportion of aneuploid cells. Data were from three male ESC lines (#S2, #S3 and #S7). More than 40 mitosis phases were counted for each group. (D) Representative images of E13.5 chimeric mice generated from mESCs (#S2) under the S, N/2i and S/2i conditions. mCherry signals represents chimeric cells in the whole embryo and Oct4-EGFP represents chimeric PGCs in the gonad. Note that S/2i-mESCs showed a greatly enhanced chimerism, whereas N/2i-mESCs did not show a promotive effect with 2i supplemented. Scale bars, 2 mm. (E and F) Skin chimerism comparison (E) and germline chimerism comparison (F) among E13.5 chimeric embryos generated from mESCs under the three indicated conditions. Here, germline chimerism = “the percentage of Oct4-EGFP+ cells in E13.5 chimeric gonad” / “the average percentage of Oct4-EGFP+ cells in E13.5 OG2-gonads (n = 11, mean = 20.258%)”. Three male mESC lines (#S2, #S3 and #S7) were analyzed. Each dot indicates the ratio of an embryo. (G) Images of viable all-ESC mice produced by indicated mESC lines by TEC assay. Data in (C), (E) and (F) are represented as the mean ± SEM. Statistical analysis was performed using a two-tailed unpaired Welch’s *t*-test. **P < 0.01; ***P < 0.001

Recently, several studies have indicated a suppressive role of 2i, whereas others clarified that they could harness mESCs in a naïve pluripotency (Sato et al., 2004; Ying et al., 2008). In order to resolve this contradiction, two culture conditions including N/2i condition and classical serum-based condition supplemental with 2i (defined as S/2i condition and the mESCs as S/2i-mESCs) were applied and carefully analyzed to uncover the underlying differences in serum and serum-free conditions and to find which is more conducive to maintaining the developmental potential of mESCs. To begin on the same starting line, we first derived mESC lines under the conventional serum condition from blastocysts carrying the Oct4-ΔPE-EGFP (OG2) reporter, in which the EGFP gene is under the control of the promoter and distal enhancer of *Oct4*. To eliminate the influence of sex, most of the experiments of this study were done using three male mESCs lines (line #S2, #S3 and #S7), which were derived and cultured in S condition (termed as S-mESCs) possessing normal karyotypes (40, XY) and high potency (Fig. 1A–D). Then, these three lines were adapted to S/2i and N/2i conditions separately for 15 days and compared to the original cell lines after the same passaging in S condition (Fig. 1A). Apparently, mESCs cultured in three conditions all exhibited tightly packed and domed colonies and showed remarkable uniform maintenance of Oct4-EGFP (Fig. 1B). However, N/2i-mESCs showed an obvious smaller colony morphology, suggesting an impaired proliferation capability (Fig. 1B). Accordingly, EdU assays confirmed that the cell cycle was disordered in N/2i-mESCs (Fig. S1A). Worse still, these N/2i-mESCs showed severe aneuploidy and become even worse with prolonged culture time (Figs. 1C, S1B and S1C). As a comparison, these abnormalities were not noticed in S/2i-mESCs, which suggested some factors lacked in serum-free condition might account for these defects.

Considering euploidy and proliferation are important for pluripotency (Pauklin and Vallier, 2014; Gonzales et al., 2015; Boward et al., 2016; Zhang et al., 2016; Kim et al., 2018), we then performed chimera assays to detect the *in vivo* developmental potential of mESCs under N/2i and S/2i conditions. In order to determine the chimerism of candidate mESCs systematically and objectively, mESCs were further marked by globally expressed mCherry. Then, 10–15 Oct4-EGFP (OG2) and mCherry double-positive (EGFP+/mCherry+) mESCs cultured under specific conditions were separately microinjected into E3.5 blastocysts (Fig. S1D). At E13.5, the chimerism rate of skin and germline of chimeric embryos generated from individual mESC lines were determined by the proportion of fluorescent chimeric cells (Fig. S1E). In contrast to the time-consuming classical method, this fluorescence-activated cell sorting (FACS) based analysis of chimeric potency assay enabled a much quantitative and time-saving evaluation of lineage differentiation capacity (Fig. S1D–F) (details are provided in the METHODS section). Specifically, somatic chimerism is determined by the proportion of mCherry positive cells in the skin and germline chimerism is determined by the proportion of Oct4-EGFP positive primordial germ cells (PGCs) in the gonad (Fig. S1E and S1G).

Strikingly, all S/2i-mESCs showed dramatically enhanced chimerism with an average increment by 220% (#S2: 300%, #S3: 150%, #S7: 210%) and 283% (#S2: 300%, #S3: 240%, #S7: 310%) in skin and germline, respectively, compared to their corresponding S-mESCs (Fig. 1D–F). However, no functional improvement was detected in N/2i mESCs. In addition, N/2i-mESCs also showed a sharp increase in resorption rate, suggesting a development failure after post-implantation (Fig. S1H). The tetraploid embryo complementation (TEC) assay further verified that the *in vivo* development of N/2i-mESCs was greatly impaired, as no all-ESC mice could be generated (Figs. 1G and S1I; and Table 1). Collectively, we showed that S/2i-mESCs harbor a much excellent developmental potential, whereas the functional improvement driven by 2i was compromised in N/2i condition and N/2i-mESCs showed chromosomal instability, disordered proliferation and restricted developmental potentials.

**Table 1:** Summary of TEC assay

Culture condition	Passage no.	ESC lines (sex)	No. of embryos transferred	Pups born (full-term)	Breathing	Adult
S	p10	Choi et al. (XY)	63	13	10	6
S	p10	S2 (XY)	232	6	4	2
S/2i	p5 + 5^a^	S2 (XY)	165	10	8	4
N/2i	p10	Choi et al (XY)	157	5	5	0
S	~p4	Yagi et al. (XX)	432	7	5	n.d.
S	p10	S1 (XX), S6 (XX)	303	5	3	1
S/2i	p5 + 5^a^	S6 (XX)	191	8	3	1
N/2i	~p4	Yagi et al. (XX)	546	0	0	0

Full-term, mice were fully developed at the time of birth; Breathing, mice were able to establish autonomous respiration; Adult, mice lived longer than 5 weeks; n.d., not determined

^a^mESCs under S condition at p5 were adapted to the S/2i condition and cultured for another 5 passages

### Inactivated TGFβ, BMP, AKT signaling pathways in N/2i condition are improved in S/2i condition

**Figure 2:**
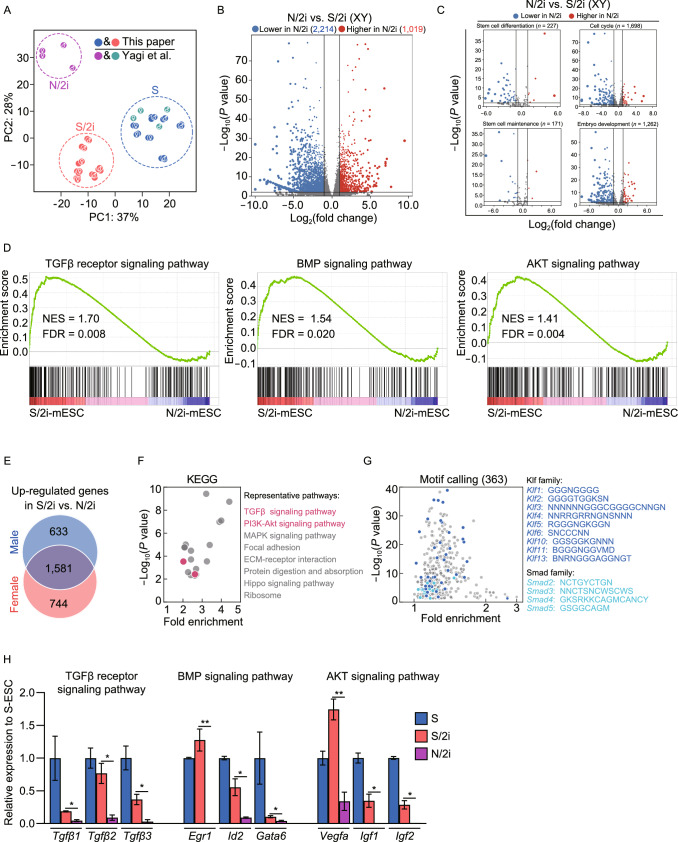
**Inactivated TGFβ, BMP, AKT signaling pathways are improved in S/2i condition**. (A) Principal component analysis (PCA) showing the transcriptome difference among S- (blue and green), S/2i- (red), and N/2i- (purple) mESCs. The sexes of mESC lines are indicated. The data for N/2i (purple) and S (green) conditions are cited from GSE84164 (Yagi et al., 2017). (B) Volcano Plot showing the global differentially expressed genes (DEGs) between male N/2i- and S/2i-mESCs. Vertical line indicates expression fold change = 2, and horizontal line indicates *P*-value = 0.01. (C) DEGs showing a dysregulation of stem cell differentiation, cell cycle, stem cell maintenance and embryo development related genes in N/2i-mESCs. These gene sets were cited from Gene Ontology Browser on MGI website (http://www.informatics.jax.org/vocab/gene_ontology). Vertical line indicates expression fold change = 2, and horizontal line indicates *P*-value = 0.01. (D) Gene-set enrichment analysis (GSEA) showing a global upregulation of genes related to TGFβ receptor signaling pathway (left, *n* = 185), BMP signaling pathway (middle, *n* = 173) and AKT signaling pathway (right, *n* = 207) in S/2i-mESCs compared to N/2i mESCs. These gene sets were cited from Gene Ontology Browser on MGI website. NES, normalized enrichment score; FDR, false discovery rate. (E) Venn diagram showing the overlap of upregulated genes in male and in female S/2i-mESCs versus N/2i-mESCs. The list of 1,581 genes are shown in Data S2. Two male and two female cell lines were used in this test. Fold change > 2, *P* < 0.01. (F) KEGG pathways enrichment analysis of the 1,581 overlapping upregulated genes, as indicated in Fig. 2E. Representative pathways are shown. (G) Calling of significantly enriched motifs (363) from the 1,581 overlapping upregulated genes caused by S/2i, as indicated in Fig. 2E. Representative motifs of Klf and Smad family are shown. (H) Representative genes related to TGFβ receptor, BMP and AKT signaling pathway were consistently downregulated in N/2i-mESCs. Data are represented as the mean  ±  SEM. Statistical analysis was performed using a two-tailed unpaired Welch’s *t*-test. *P < 0.05; **P < 0.01

To uncover the potential molecular mechanism attributing to the restricted developmental potential of N/2i-mESCs as compared to S/2i-mESCs, we performed RNA sequencing, processing and analysis. Principal component analysis (PCA) convincingly showed that the transcriptome of N/2i-mESCs was quite different from that of S/2i-mESCs (Fig. 2A). Differentially expressed genes (DEGs) analysis showed that 3,233 genes were significantly changed in male N/2i-mESCs compared with male S/2i-mESCs, of which more genes (2214) were downregulated and the others (1,019) were upregulated (Fig. 2B; Data S1). A similar result was detected in female mESCs (Fig. S2A). In addition, many key biological processes critical for pluripotency such as stem cell differentiation, cell cycle, stem cell maintenance and embryo development were mainly downregulated in N/2i-mESCs, indicating that N/2i- and S/2i-mESCs are distinct in many aspects (Figs. 2C and S2B). Strikingly, we found that the expression of housekeeping genes such as glyceraldehyde-3-phosphate dehydrogenase (*Gapdh*), tubulin beta 5 class I (*Tubb5*), actin beta (*Actb*) and hypoxanthine guanine phosphoribosyl transferase (*Hprt*) were remarkably decreased in N/2i-mESCs (Fig. S2C). 5-ethynyl uridine (5-EU) incorporation assay further indicated the global transcription activity in N/2i-mESCs was lower than that of S- and S/2i-mESCs (Fig. S2D).

As a natural biological product, serum contains many components including macromolecules represented by growth factors, which have a wide range of effects on mESCs’ characteristics including cell proliferation and cell differentiation, etc. Thus, certain growth factors in serum might be responsible for the pluripotency improvement of mESCs induced by S/2i as compared to N/2i condition. To figure out which factor(s) might play this key role, we performed a gene set enrichment analysis (GSEA) of multiple critical signaling pathways (Figs. 2D and S2E). The result showed that TGFβ, BMP and AKT signaling pathways were remarkably enhanced in S/2i-mESCs. Meanwhile, we analyzed the 1581 genes that were specifically upregulated in S/2i mESCs of both sexes by gene ontology analysis (Fig. 2E; Data S2), and found that TGFβ and PI3K-Akt signaling pathways were significantly enriched (Fig. 2F). Moreover, many of these upregulated genes were targets of Klf and Smad family members as identified by motif calling analysis (Fig. 2G). Notably, Smad family members are well-known downstream effectors of BMP signaling pathways (Ying et al., 2003; Xu et al., 2008; Morikawa et al., 2016) (Fig. S2F), and Klf family can be regulated by Smad1/5 (Fig. S2G). Thus, the motif-calling analysis highly pointed out a big difference in BMP signaling pathway between N/2i- and S/2i-mESCs. In addition, the expression of representative genes of the above three signaling pathways were indeed downregulated in N/2i-mESCs and restored in S/2i-mESCs (Fig. 2H).

Taken together, TGFβ, BMP and AKT signaling pathways were inactivated in N/2i-mESCs. As a comparison, these pathways were restored in S/2i-mESCs which might be responsible for the observed developmental improvement (Fig. 1D–G).

### BMP4 safeguards the chromosomal integrity and preserves the developmental potential of mESCs

**Figure 3:**
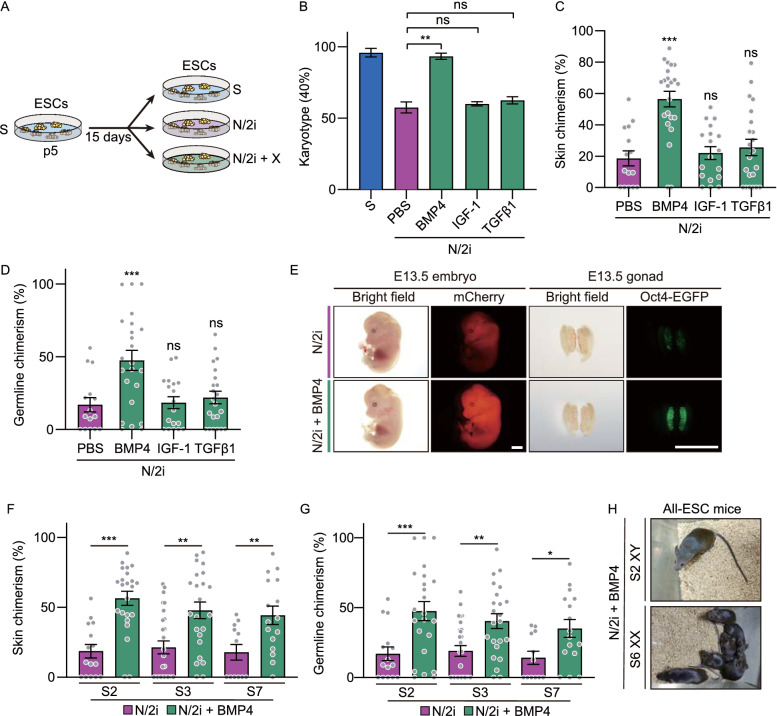
**BMP4 safeguards the chromosomal integrity and preserves the developmental potential of mESCs**. (A) Strategy for comparing the same mESC line cultured under different conditions. p, passage. X indicates candidate growth factors used in Table 2. (B) Karyotyping validation of mESCs cultured under indicated conditions. Note that BMP4 can effectively preserve the chromosomal integrity of N/2i-mESCs. Three male mESC lines (#S2, #S3 and #S7) were analyzed. (C and D) Skin (C) and germline (D) chimerism comparison between N/2i + PBS-mESCs (control) and mESCs under indicated culture conditions. A male mESC line #S2 was analyzed. Each dot indicates the ratio of an embryo. (E) Representative images of E13.5 chimeric mice generated from mESCs line #S2 under the N/2i and N/2i + BMP4 conditions. mCherry signals in the whole embryo and Oct4-EGFP + PGCs in the gonad are shown. Scale bars, 2 mm. (F and G) BMP4 greatly enhanced the developmental potential of mESCs as confirmed by skin (F) and germline (G) chimerism comparision. (H) TEC assay showing that BMP4 supplement supports the generation of adult all-ESC mice from N/2i-mESCs of both sexes. Data are represented as the mean ± SEM in (B–D), (F) and (G). Statistical analysis was performed using a two-tailed unpaired Welch’s *t*-test. *P < 0.05; **P < 0.01; ***P < 0.001. ns, not significant

The above results suggested that we might be able to preserve the developmental potential of mESCs by activating specific pathways. To this end, we performed a small screening of growth factors that have been proved to activate TGFβ, BMP or AKT signaling pathways separately. Specifically, mESCs were cultured in N/2i condition supplemented with specific growth factor for 15 days for analysis and comparison (Fig. 3A). Growth factors including BMP2/4/7, Activin A, IGF-1, TGFβ1, bFGF, EGF and FGF4 were tested, and PBS (phosphate-buffered saline) was set as a control (Table 2). Considering the importance of chromosomal integrity in pluripotency maintenance, we then asked whether the addition of candidate growth factor could help to sustain euploidy in N/2i condition. Finally, BMP4 was identified with the best effect followed by BMP7 and BMP2, which indicates BMP signaling pathway is essential for the maintenance of chromosomal integrity in N/2i condition (Fig. 3B; Table 2). Notably, this protective effect continued to work during the long-term propagation of mESCs (Fig. S3A and S3B). Moreover, N/2i + BMP4-mESCs exhibited fewer γH2A.X enriched loci, which indicated a reduced DNA damage after BMP4 supplement (Fig. S3C). The reactivation of BMP signals could further restore the proliferation capability of N/2i-mESCs (Fig. S3D). Specifically, the impaired cell cycle was recovered with fewer cells stuck in the G_1_ phase and shortened cell cycle duration (Fig. S3E and S3F).

**Table 2:** Karyotyping of mESCs adapted to N/2i condition supplemented with candidate factors

Rank	Growth factors	Concentration (ng/mL)	Karyotype (40, %)			
			S2	S3	S7	Mean
1	BMP4	10	80.00	82.50	85.00	82.50
2	BMP7	10	62.50	67.50	55.00	61.67
3	BMP2	10	55.00	47.50	52.50	51.67
4	Tgfβ1	2	50.00	55.00	47.50	50.83
5	IGF-1	10	45.00	47.50	60.00	50.83
6	Activin A	20	42.50	60.00	45.00	49.17
7	bFGF	10	52.50	37.50	45.00	45.00
8	PBS^a^	N/A	55.00	37.50	35.00	42.50
9	EGF	10	35.00	42.50	42.50	40.00
10	FGF4	25	47.50	40.00	32.50	40.00

^a^Blank control; N/A, Not Applicable

Given the important role of BMP signals in maintaining the euploidy and self-renewal capability of mESCs, we then investigated whether activation of BMP signals can functionally improve the developmental potential of N/2i-mESCs. Strikingly, the addition of BMP4 to N/2i mESCs significantly improved the chimerism by 180% in the skin (Fig. 3C and 3E) and 150% in the germline (Fig. 3D and 3E). Meanwhile, this improvement was consistent across all tested mESC lines (Fig. 3F and 3G). In addition, the abnormally higher resorption rate of N/2i-mESCs significantly decreased with BMP4 supplemented (Fig. S3G). Finally, we performed the TEC assay to test the autonomous developmental potential of these mESCs. In sharp contrast to N/2i-mESCs, N/2i + BMP4-mESCs of both sexes could efficiently generate live all-ESC mice (Figs. 3H and S1I; Tables 1 and 3). Taken together, our results showed that BMP4 activation can preserve the developmental potential and safeguard the chromosomal integrity of mESCs.

**Table 3:** Results of N/2i + BMP4- and N/2i-mESCs TEC assay

Culture condition	Passage no.	mESC lines (sex)	No. of embryos transferred	Pups born (Full-term)	Breathing	Adult
N/2i + BMP4	p5+5^a^	S2 (XY)	120	15	4	1
N/2i	p5+5^a^	S2 (XY)	180	8	1	0
N/2i + BMP4	p5+5^a^	S6 (XX)	135	22	17	6
N/2i	p5+5^a^	S6 (XX)	200	0	0	0

Full-term, mice were fully developed at the time of birth; Breathing, mice were able to establish autonomous respiration; Adult, mice lived longer than 5 weeks

^a^mESCs under S condition at p5 were adapted to the N/2i + BMP4 or N/2i condition and cultured for another 5 passages

### Ube2s and Chmp4b are essential for BMP4-mediated chromosomal integrity safeguarding and pluripotency maintenance

**Figure 4:**
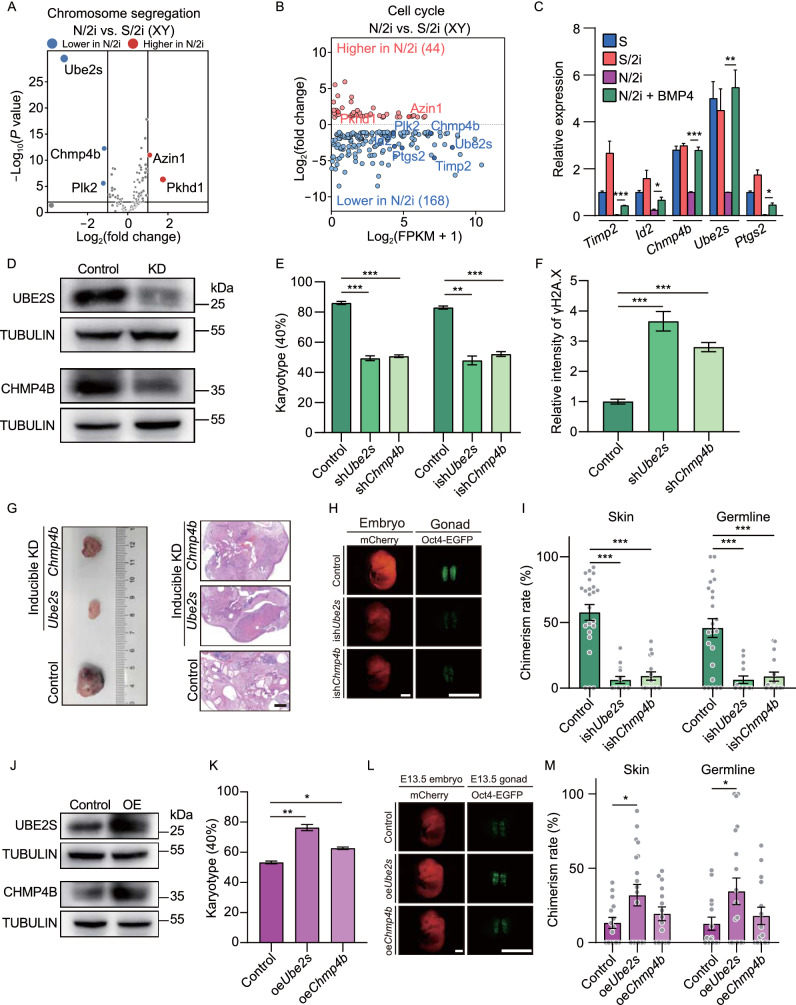
***Ube2s*** **and** ***Chmp4b*** **are essential for BMP4-mediated chromosomal integrity safeguarding and pluripotency maintenance**. (A) Volcano plot showing chromosome segregation-related DEGs between male N/2i- and S/2i-mESCs. Vertical lines indicate expression fold change = 2, and horizontal lines indicate *P*-value = 0.01. Basements of *Ube2S*, *Chmp4b*, *Azin1*, *Plk2* and *Phkd1* were 332.24, 115.55, 40.45, 18.70 and 0.39, respectively. (B) MA (ratio intensity) plot showing 212 dysregulated cell cycle related genes in N/2i-mESCs compared to S/2i-mESCs. Certain upregulated and downregulated genes are marked in red and blue, respectively. The list of 1,698 cell cycle-related genes are cited from MGI gene ontology (Ashburner et al., 2000). (C) RT-qPCR analysis showing the relative RNA level of the indicated cell cycle related genes in S-, S/2i-, N/2i- and N/2i + BMP4-mESCs. *Hprt* was set as an endogenous control. Three male mESC lines (#S2, #S3, and #S7) were tested with 3 biological replicates. (D) Western blot validated the decrease of Ube2s and Chmp4b protein levels after shRNA mediated gene knockdown in mESCs. mESC line #S2 was used in this test and cultured under N/2i + BMP4 condition. The scramble shRNA transfected mESCs were set as the control. KD, knockdown. (E) Karyotype analysis demonstrated that downregulation of *Ube2s* or *Chmp4b* caused severe chromosomal aberrations. More than 40 mitosis phases were counted for each group. *n* = 3 biological replicates. (F) Quantification of γH2A.X signal in control-, sh*Chmp4b*- and sh*Ube2s*-mESCs related to Fig. S5F. mESC line #S2 was used in this test. *n* = 35 single cell nuclear. (G) Knockdown of *Ube2s* or *Chmp4b* in mESCs for 5 passages greatly impaired the formation and differentiation of teratomas. Teratomas were dissected for HE staining. mESC line #S2 was used in this test. The scramble ishRNA (inducible shRNA) transfected mESCs were set as the control. KD, knockdown. Scale bar, 1 mm. (H) Representative images of E13.5 chimeric mice generated by indicated mESCs. Chimeric mice generated by scramble ishRNA mESCs were set as the control. mESC line #S2 was used in this test. Scale bars, 2 mm. (I) Knockdown of *Ube2s* or *Chmp4b* for 5 passages caused a sharp decrease in both skin and germline chimerism. Each dot indicates the ratio of an embryo related to Fig. 4H. (J) Western blot validation of Ube2s and Chmp4b protein levels in indicated mESCs. mESC line #S2 was used in this test and cultured under N/2i condition. The empty vector transfected mESCs were set as the control. OE, overexpression. (K) Karyotype analysis demonstrated that upregulation of *Ube2s* or *Chmp4b* could rescue chromosomal aberrations in N/2i-mESCs. More than 40 mitosis phases were counted for each group. *n* = 3 biological replicates. (L) Representative images of E13.5 chimeric mice generated by indicated mESCs. Scale bars, 2 mm. (M) Overexpression of *Ube2s* or *Chmp4b* in N/2i-mESCs led to an increasement in chimerism. Each dot indicates the ratio of an embryo related to Fig. 4L. Data are represented as the mean ± SEM in (C), (E), (F), (I), (K) and (M). Statistical analysis was performed using a two-tailed unpaired Welch’s *t*-test. *P < 0.05; **P < 0.01; ***P < 0.001. ns, not significant

Considering the aneuploidy and restricted proliferation in N/2i-mESCs, we next focused on chromosome segregation and cell cycle related genes that were dysregulated in N/2i-mESCs as compared to S/2i-mESCs which might be the potential downstream targets of the BMP signal pathway (Fig. 4A and 4B; Table 4). Here, we showed that most of these genes exhibited a downward trend, represented by ubiquitin-conjugating enzyme E2S(*Ube2s*), charged multivesicular body protein 4B (*Chmp4b*)*,* polo like kinase 2 (*Plk2*)*,* prostaglandin-endoperoxide synthase 2 (*Ptgs2*)*,* tissue inhibitor of metalloproteinase 2 (*Timp2*) and inhibitor of DNA binding 2 (*Id2*). The RT-qPCR analysis further showed that five genes including *Timp2*, *Id2*, *Chmp4b*, *Ube2s* and *Ptgs2* were abnormally expressed only in N/2i-mESCs. Meanwhile, these genes could be restored with BMP4 supplemented, in which *Ube2s* and *Chmp4b* were the most obvious (Figs. 4C and S4A). Besides, *Ube2s* and *Chmp4b* are closely related to the regulation of both chromosome segregation and cell cycle (Fig. 4A and 4B). In addition, these two genes can be targeted by zinc finger and BTB domain containing 7a (*Zbtb7a*) in mESCs, which was proved to be a target of BMP-SMAD signaling pathway (Fig. S4B) (Yu et al., 2020). We further confirmed that overexpression of *Zbtb7a* in N2i-mESCs could upregulate the expression of *Ube2s* (Fig. S4C). Notably, *Ube2S* can fine-tune *Sox2* expression and reinforce the self-renewal and pluripotency of mESCs (Wang et al., 2016) . Moreover, we found that BMP4 could orchestrate the histone modifications at the regulatory regions of *Ube2s* and *Chmp4b* (Fig. S4D). Specifically, an increased active H3K4me3 level at the promoter of *Ube2s* and *Chmp4b* was detected in N/2i + BMP4-mESCs when compared to that in N/2i-mESCs, whereas levels of the repressive markers such as H3K9me3 and H3K27me3 decreased (Fig. S4D). Based on these findings, *Ube2s* and *Chmp4b* might be important targets that functioned in BMP4-mediated improvements on N/2i-mESCs.

**Table 4:** Chromosome segregation related genesets

Term	ID	No. of genes	No. of significantly changes genes
DNA topoisomerase type II activity	GO:0003918	4	0
Condensin complex	GO:0000796	8	0
Anaphase-promoting complex	GO:0005680	21	**1 (*Ube2s*)**
Kinetochore microtubule	GO:0005828	6	0
Cohesin complex	GO:0008278	10	0
Spindle assembly checkpoint signaling	GO:0071173	38	0
Centrosome duplication	GO:0051298	76	**4 (*Pkhd1*, *Plk2*, *Chmp4b*, *Azin1*)**

All of these genesets were cited from MGI database (http://www.informatics.jax.org/vocab/gene_ontology)

Given both *Ube2s* and *Chmp4b* are essential for ensuring the precise regulation of cell cycle progression and accurate chromosome segregation, we asked whether altering the expression of *Ube2s* and *Chmp4b* in mESCs would result in similar deficiencies observed in N/2i-mESCs. We then generated *Ube2s-* and *Chmp4b-*deficient mESCs in N/2i + BMP4 condition through shRNA mediated gene knockdown, and the knockdown of these genes was validated by immunoblotting and RT-qPCR analysis (Figs. 4D and S5A). These sh*Ube2s-* and sh*Chmp4b-*mESCs were similar to control mESCs in Oct4-EGFP maintaining but exhibited a markedly smaller colony size after propagation, which indicated an impaired proliferation (Fig. S5B). The EdU incorporation assay further confirmed the significantly disordered cell cycle and extended cell cycle duration especially in sh*Ube2s*-mESCs (Fig. S5C and S5D). In addition, sh*Ube2s*- or sh*Chmp4b*-mESCs showed severe aneuploidy and DNA damage. Notably, these defects could not be rescued under BMP4 containing condition, which indicates that *Ube2s* and *Chmp4b* are the key targets of BMP4 (Figs. 4E, 4F, S5E and S5F).

We then evaluated whether alteration of these two genes would further affect the developmental potential of mESCs through teratoma and chimera assay. In order to avoid an irreversible damage on cell proliferation by the constitutive gene knockdown, we generated inducible *Ube2s*- and *Chmp4b*-deficient mESCs (termed as ish*Ube2s*-mESCs and ish*Chmp4b*-mESCs) by applying the Dox-inducible shRNA system (Fig. S5A and S5G). Specifically, these mESCs were firstly cultured under the Dox-containing condition for 5 passages and then used for further analyses without Dox induction. The result clearly indicated teratomas generated by ish*Ube2s*- or ish*Chmp4b*-mESCs were much smaller than that generated by control mESCs (Fig. 4G), which indicated the proliferation of these mESCs was severely impaired. Moreover, these tiny teratomas were poorly differentiated when compared to the control teratoma with distinct three-germ-layer differentiated cells (Fig. 4G), indicating that short-term knockdown of *Ube2s* or *Chmp4b* was enough to cause aneuploidy which further destroyed the differentiation ability of mESCs (Fig. 4E). Strikingly, after applying ish*Ube2s*-mESCs or ish*Chmp4b*-mESCs as donor cells in the chimera assay, only a few chimeric embryos could be obtained and these embryos presented dramatic lower percentage of mCherry+ and Oct4-EGFP+ cells in skin and gonad, respectively (Fig. 4H and 4I). Besides, ish*Ube2s*- and ish*Chmp4b*-mESCs showed an abnormally increased resorption rate (1.95-fold and 2.0-fold) similar to N/2i-mESCs (Fig. S1H and S5H). For further comparison, *Ube2s*- and *Chmp4b*-overexpressed N/2i-mESCs were generated and analyzed (Figs. 4J and S5I). Specifically, these two cell lines exhibited a higher percentage of euploid cells and a recovered proliferation capacity in the N/2i condition (Figs. 4K and S5J). Notably, *Ube2s*-overexpressed N/2i-mESCs showed an enhanced *in vivo* developmental potential and a reduced resorption rate as confirmed by chimera assay, while *Chmp4b* overexpression presented a relatively modest effect (Figs. 4L, 4M and S5K).

Taken together, these data demonstrated that *Ube2s* and *Chmp4b* are critical downstream targets of BMP4, both of which are essential for BMP4-mediated chromosomal integrity safeguarding, normal proliferation capacity and developmental potential maintenance of mESCs.

### BMP4 can restore the dysregulated transcriptome and long-termly preserve the pluripotency of N/2i-mESCs

**Figure 5:**
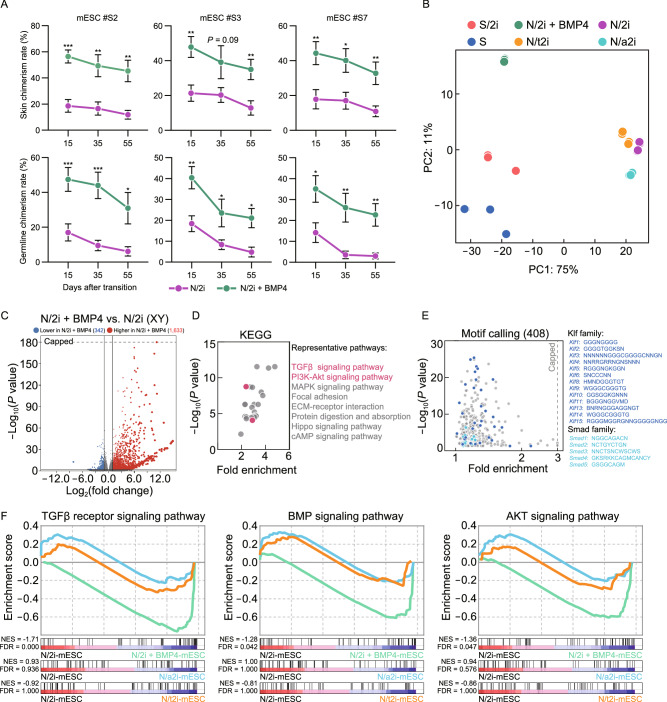
**BMP4 can restore the dysregulated transcriptome and long-termly preserve the pluripotency of N/2i-mESCs**. (A) Skin (upper) and germline (lower) chimerism comparison indicated that BMP4 could sustain the developmental potential of N/2i-mESCs during long-term culture. The horizontal axis represented days after transition to indicated culture conditions. Three male mESC lines (#S2, #S3, and #S7) were tested. Data are represented as the mean ± SEM. Statistical analysis was performed using a two-tailed unpaired Welch’s *t*-test. *P < 0.05; **P < 0.01; ***P < 0.001. (B) PCA showing the transcriptome difference among S-, S/2i-, N/2i + BMP4-, N/2i-, N/a2i- and N/t2i-mESCs. (C) Volcano plot showing the global differentially expressed genes between N/2i + BMP4- and N/2i-mESCs. Vertical line indicates expression fold change = 2, and horizontal line indicates *P*-value = 0.01. (D) KEGG pathways enrichment analysis of 1,633 upregulated genes caused by BMP4, as indicated in Fig. 5C. Representative pathways are shown. (E) Calling of significantly enriched motifs (408) from the 1,633 upregulated genes caused by BMP4, as indicated in Fig. 5C. Representative motifs of Klf and Smad family are shown. (F) GSEA showing the global change of TGFβ receptor signaling pathway (left, *n* = 185), BMP signaling pathway (middle, *n* = 173) and AKT signaling pathway (right, *n* = 207) in indicated mESCs compared to N/2i-mESCs. These gene sets were cited from Gene Ontology Browser on MGI website. NES, normalized enrichment score. FDR, false discovery rate

Nowadays, the application of pluripotent stem cells is a major part of regenerative medicine, whereas diversity operations like genetic engineering inevitably require prolonged *in vitro* passaging. How to preserve the quality of these cells during long-term culture remains to be solved. Here, we were glad to share that the N/2i + BMP4 condition could preserve the developmental potential of mESCs with prolonged culture time. After culturing mESCs for about 20 passages, all the three mESC lines (#S2, #S3 and #S7) maintained remarkably higher chimerism in both skin and germline. Strikingly, the chimerism of chimeric embryos generated by N/2i + BMP4-mESCs after 55 days of culture was even higher than those of N/2i-mESCs cultured for only 15 days (Figs. 5A, S6A and S6B). Notably, this pluripotency preservation was much important for mESCs’ germline transmission ability, as chimeric embryos generated by prolonged cultured N/2i-mESCs almost lost Oct4-EGFP signals in the gonad (Figs. 5A and S6B).

Considering the enhanced developmental potential of mESCs under N/2i + BMP4 condition, we next performed RNA-Seq analysis to explore the effect of BMP4 on transcriptome. Both PCA and hierarchical clustering plots indicated that the transcriptome of N/2i + BMP4-mESCs was quite different from that of N/2i-mESCs, and was much similar to those under serum condition (both S- and S/2i-mESCs) (Figs. 5B and S6C). Importantly, many key genes critical for pluripotency that were abnormally downregulated in N/2i-mESCs were successfully recovered only in the BMP4 supplemented condition (Figs. 2C and S6D). DEG analysis further showed that 1,633 genes were upregulated in total 1,975 significantly changed genes influenced by BMP4 under N/2i condition (Fig. 5C; Data S3). Besides, gene ontology on the KEGG (Kyoto Encyclopedia of Genes and Genomes) pathway of the 1,633 upregulated genes suggested TGFβ and PI3K-Akt signaling pathways were significantly enriched, and many of these genes were downstream targets of Klf and Smad family members as identified by motif calling analysis (Fig. 5D and 5E). This finding was much similar to that observed in S/2i-mESCs versus N/2i-mESCs (Fig. 2F and 2G), which further confirmed a critical role of BMP4 in serum system but lacked in chemically defined N/2i condition. Moreover, GSEA demonstrated that impaired TGFβ, BMP and AKT signal pathways in N/2i-mESCs were significantly restored with BMP4 supplemented (Fig. 2D and 5F).

In summary, BMP4 largely restored critical genes and signal pathways dysregulated under chemically defined condition and could sustain the enhanced developmental potential of mESCs during long-term culture.

### N/2i + BMP4-mESCs exhibit distinct pluripotent features compared with N/a2i- and N/t2i-mESCs

**Figure 6:**
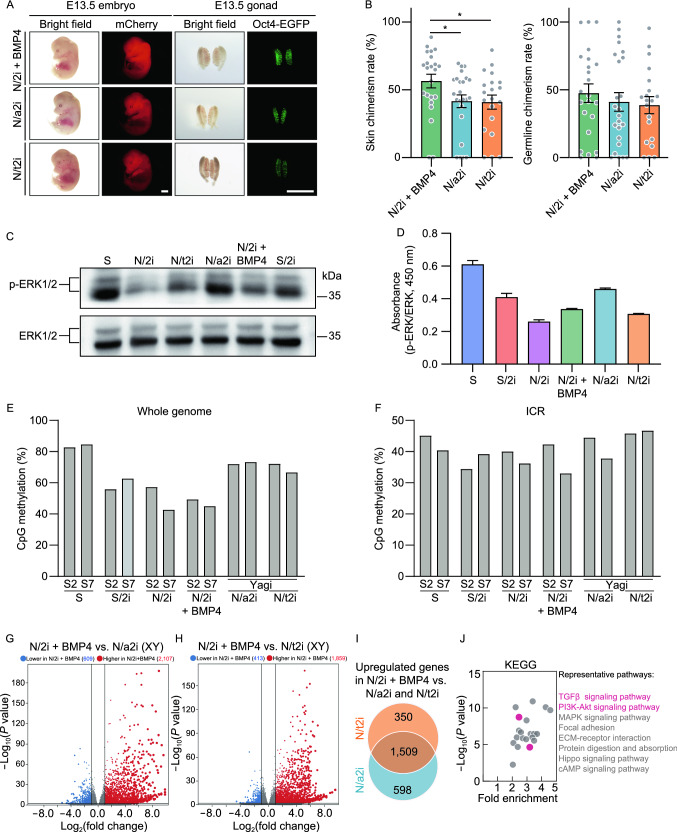
**N/2i + BMP4-mESCs exhibit distinct pluripotent features compared with N/a2i- and N/t2i-mESCs**. (A) Representative images of E13.5 chimeric mice generated from mESCs line #S2 under the N/2i + BMP4, N/a2i and N/t2i conditions. Scale bars, 2 mm. (B) Skin (left) and germline (right) chimerism comparison of mESC line #S2 under indicated culture conditions. N/2i + BMP4-mESCs have an increased skin chimerism compared with N/a2i- and N/t2i-mESCs. Each dot indicates the ratio of an embryo. (C and D) Western blot (C) and ELISA assay (D) of ERK1/2 and p-ERK1/2 in mESC line #S2 under indicated culture conditions. The result showed that BMP4 did not greatly attenuate the repression of p-ERK1/2. (E and F) Whole genome bisulfite sequencing (WGBS) showing the whole genome (E) and imprinting control region (ICR) (F) methylation levels in indicated mESCs. Data for N/a2i and N/t2i conditions are cited from GSE84164 (Yagi et al., 2017). (G) Volcano plot showing the DEGs between male N/2i + BMP4- and N/2i-mESCs. Vertical line indicates expression fold change = 2, and horizontal line indicates *P*-value = 0.01. (H) Volcano plot showing the DEGs between male N/2i + BMP4- and N/t2i-mESCs. Vertical line indicates expression fold change = 2, and horizontal line indicates *P*-value = 0.01. (I) Venn diagram showing the overlap of upregulated genes in male N/2i + BMP4-mESCs versus N/a2i- and N/t2i-mESCs. The list of 1,509 genes are shown in Data S4. Fold change > 2, *P* < 0.01. (J) KEGG pathways enrichment analysis of the 1,509 overlapping upregulated genes caused by BMP4, as indicated in Fig. 6I. Representative pathways are shown. Data are represented as the mean ± SEM in (B) and (D). Statistical analysis was performed using a two-tailed unpaired Welch’s *t*-test. *P < 0.05

In this study, we have verified that N/2i + BMP4-mESCs had an enhanced developmental potential compared to N/2i-mESCs. As N/a2i- and N/t2i-mESCs also presented ground-state pluripotency of mESCs under the serum-free condition (Choi et al., 2017; Yagi et al., 2017), we further tested which of these three improvements showed the best maintenance of pluripotency. Then, the mESC line #S2 was adapted to N/2i + BMP4, N/a2i and N/t2i conditions separately for 15 days for further comparisons. The adapted mESCs cultured in these three serum-free conditions all exhibited uniform maintenance of Oct4-EGFP. However, N/a2i-mESCs showed a smaller colony morphology with a relatively rough surface (Fig. S7A). Karyotype analysis demonstrated that all these three conditions could sustain chromosomal integrity of mESCs in general (Fig. S7B and S7C), quite different from that observed in N/2i-mESCs (Fig. S1B). In terms of differentiation capacity, these three conditions showed certain differences. Notably, N/2i + BMP4-mESCs showed the highest *in vivo* developmental potential compared to N/a2i-mESCs or N/t2i-mESCs (Fig. 6A and 6B).

It is worth noting that N/a2i or N/t2i condition improved the quality of mESCs mainly through attenuating the repression of ERK1/2 cascade and regulating DNA methyltransferases and cofactors (Fig. S7D) (Shimizu et al., 2012; Yagi et al., 2017). In contrast, BMP4 hardly influenced the phosphorylation of ERK1/2, which was validated by both immunoblotting assay and ELISA (enzyme-linked immunosorbent assay) (Fig. 6C and 6D). And the expression of DNA methylation related genes was not greatly changed after BMP4 addition in N/2i condition (Fig. S7D). Moreover, whole-genome bisulfite sequencing (WGBS) indicated BMP4 had no effect on DNA methylation recovery. The result showed that S/2i, N/2i and N/2i + BMP4 all caused an obvious genome-wide hypomethylation in mESCs, whereas attenuating the repression of MEK sustained the parental differentially methylated regions (DMRs) as reported (Fig. 6E) (Choi et al., 2017; Yagi et al., 2017). Interestingly, the imprinting control regions (ICRs) methylation did not show a great alteration in all groups after 15 days of culture condition transition (Fig. 6F).

To further figure out the transcriptional differences among N/2i + BMP4-, N/a2i- and N/t2i-mESCs, we performed the RNA-seq analysis. The result suggested gene expression changes caused by N/a2i or N/t2i were relatively moderate as compared to those by N/2i + BMP4 (Figs. 5C, S7E and S7F; Data S3). And N/2i-, N/a2i- and N/t2i-mESCs showed a much similar transcriptome with a relative inactive global transcription level, which was distinct from that of N/2i + BMP4-mESCs (Figs. 5B, 6G, 6H, S6C, S7G and S7H). Strikingly, the expression level of pluripotency related key genes was still insufficient in both N/a2i- and N/t2i-mESCs (Fig. S6D). Worse still, attenuating MEK suppression could not recover the impaired TGFβ, BMP and AKT signal pathways in mESCs (Figs. 5F, 6I, 6J; Data S4).

Taken together, N/2i + BMP4-mESCs exhibit a better pluripotency compared with N/a2i- and N/t2i-mESCs and we recommend the N/2i + BMP4 condition for the best quality of mESCs under serum-free system.

## Discussion

ESCs, which can differentiate into all the specialized cell types of an adult organism and thus constitute an invaluable platform for modeling developmental processes and diseases, have the capacity for unlimited self-renewal and pluripotency properties. In recent years, a number of culture conditions, including conventional serum and chemically defined serum-free N2B27 system, have been established to support *in vitro* maintenance of pluripotency (Ying et al., 2008; Hackett and Surani, 2014). Here, we identified the BMP signaling pathway is deficient in the chemically defined 2i condition (N/2i), which greatly impairs the developmental potential of mESCs. Addition of BMP4 in N/2i condition can restore the expression of *Ube2s* and *Chmp4b*, which further safeguards chromosomal integrity and largely promotes *in vivo* differentiation potential of mESCs. In addition, this effect of BMP4 continues to work during the long-term culturing of mESCs. Notably, N/2i + BMP4-mESCs possess a better pluripotency over N/a2i- and N/t2i-mESCs. Together, our study reveals a valid role of BMP4 in harnessing the functional pluripotency capacity of mESCs under the chemically defined culture system.

Many criteria have been developed and applied to determine the quality of mESCs by various methods, including karyotyping, transcription, epigenomic modification as well as *in vitro* and *in vivo* differentiation assay (Nichols and Smith, 2009; Leitch et al., 2013; Kolodziejczyk et al., 2015; Guo et al., 2018). Notably, pluripotency is determined not only by the expression of marker genes or an ICM-like epigenomic status but also by an active and appropriate differentiation potential, especially the ability to generate PGCs, which has been viewed as the gold standard (Bradley et al., 1984; Gardner, 1998; Tam and Rossant, 2003; Hackett and Surani, 2014). Here, by using mESCs double-labeled with Oct4-ΔPE-EGFP and globally expressed mCherry, we were able to quantitatively determine the somatic chimerism (mCherry+) and germline ability (EGFP+) of mESC candidates within 2 weeks. And this assay also allows sufficient comparisons of target cell lines to permit more accurate and sensitive conclusions to be drawn. After carefully comparing chimeric embryos generated by N/2i and S/2i conditions, we found the developmental potential of N/2i-mESCs was restricted. However, this impairment is not simply attributed to the MEKi or GSK3βi treatment, as S/2i-mESCs showed a remarkably higher somatic and germline chimerism. The transcriptome analysis and growth factor screen assay further uncovered the BMP signal is deficient in N/2i condition, and the BMP4 supplement could preserve euploidy, reset the self-renewal capacity, and promote pluripotency of mESCs during prolonged culturing.

As an important growth factor, BMP4 has been reported to sustain self-renewal and preserve multilineage differentiation of mESCs in combination with LIF in the serum-free condition (Ying et al., 2003). Later, 2i (CHIR99021 and PD0325901) condition was developed which could greatly improve the derivation efficiency and keep mESCs in a highly homogeneous state (Ying et al., 2008). In this culture system, the requirement for serum/BMP was replaced by PD0325901, a potent and selective MEK inhibitor that was claimed to have no distinct side effects. However, we demonstrated an irreplaceable role for activating BMP signals in the chemically defined serum-free condition, which greatly helps to safeguard the chromosomal integrity and pluripotency through regulating specific downstream targets *Ube2s* and *Chmp4b*. Notably, these functions are different from the previous role of BMP4 in inhibiting differentiation genes and sustaining the self-renewal of mESCs in collaboration with STAT3 (Ying et al., 2003). More recently, Wu reported Activin A and BMP4 signaling could expand potency of mESCs under serum-free condition (Wu et al., 2020), in which the MEK1/2 inhibitor was replaced by Activin A. However, it has been proved that just weakening MEK suppression (either by N/t2i or N/a2i) could maintain pluripotency mainly through fine-tuning DNA methylation in mESCs (Choi et al., 2017; Yagi et al., 2017).

Here, our study showed that BMP4 supplement alone could greatly improve the self-renewal and developmental potential of N/2i-mESCs. More importantly, the *in vivo* developmental potential of these N/2i + BMP4-mESCs was better than that of N/a2i-mESCs or N/t2i-mESCs. This function of BMP4 works mainly through regulating its target genes *Ube2s* and *Chmp4b*, partially by fine-tuning the active and repressive histone modifications at their promoters. In addition, BMP4 also regulates other key genes and pathways critical for pluripotency, but it does not work through directly antagonizing the effects of MEK inhibitors or through maintaining the expression of methylation related genes and global DNA methylation levels. However, we could not rule out the possibility that BMP signals could also act downstream of phospho-ERK to block mESC commitment. Besides, how BMP4 precisely orchestrates histone modifications at the promoter regions of *Ube2s* and *Chmp4b* remains to be studied. Collectively, these findings emphasize that BMP4 plays multiple essential roles in regulating pluripotency.

Euploidy is one of the most basic requirements of high-quality pluripotent stem cells. Once cells become aneuploid, it is almost impossible to recover naturally even the N/2i-mESCs were returned to be cultured in serum system for a prolonged time (Fig. S7I). Of note, although aneuploidy is usually to be a hallmark of cancer, aneuploid mESCs did exhibit impaired differentiation capacity (Zhang et al., 2016), which was also confirmed in our ish*Ube2s*/*Chmp4b-*mESCs (Fig. 4H and 4I). Some studies reported that hypomethylation could cause chromosomal instability (Sheaffer et al., 2016) and reduced MEK inhibition could preserve genomic stability (Di Stefano et al., 2018). However, it was also shown that the hypomethylation caused by DNA methyltransferase 1 (*Dnmt1*), 3A (*Dnmt3a*) and 3B (*Dnmt3b*) triple-knockout did not cause karyotype abnormalities (Choi et al., 2017). Here, we suggested that hypomethylation caused by 2i addition would not cause obvious aneuploidy in serum condition for a prolonged propagation (Fig. S1C), whereas it is the deficient BMP-*Ube2s*/*Chmp4b* accounts for aneuploidy. Unlike the irreversibility of aneuploidy, DNA methylation can exhibit dynamic changes. In line with this notion, hypomethylation lasting for approximately 1 week in mouse PGCs and several months in human PGCs does not cause chromosomal abnormalities, but further supports pluripotency remodeling (Gkountela et al., 2015; Guo et al., 2015; Tang et al., 2015). The hypomethylation caused by MEKi could be restored by culturing mESCs in S condition for a few days (Habibi et al., 2013). However, this DNA methylation recovery failed to occur in ICRs as prolonged treatment with MEKi would result in irreversible loss of imprints that compromises the developmental potential of mESCs of both sexes (Choi et al., 2017). DNA hypomethylation in ICM of a blastocyst only exists for a very short period, whereas DNA hypomethylation caused by MEKi can be maintained and even decreases during prolonged *in vitro* propagation (Smith et al., 2012; Leitch et al., 2013). Interestingly, we found a global hypomethylation and an even lower ICR methylation level did not greatly affect S/2i-mESCs’ ability in generating all-ESC mice (Table 5), whereas it is the chemically defined N/2i condition compromises the differentiation potentials. Thus, it is premature to attribute the reduced developmental potential of N/2i-mESCs entirely to the erosion of genomic imprints. The transcriptional dysregulation, aneuploidy and impaired proliferation caused by the N/2i condition should also be considered.

**Table 5:** Summary of methylation level and TEC assay

mESC lines (sex)	Methylation ratio (%)		No. of embryos transferred	Pups born (full-term)	Breathing	Adult
	Whole genome	ICR				
K3 (XY)	52.80	23.14	106	7	7	2
K7 (XX)	8.18	3.59	90	4	2	1

mESC lines K3 and K7 were derived and cultured in S/2i condition until p8 and detected by whole genome bisulfite sequencing

Full-term, mice were fully developed at the time of birth; Breathing, mice were able to establish autonomous respiration; Adult, mice lived longer than 5 weeks

In summary, our study found the irreplaceable role of BMP4 in sustaining chromosomal integrity and pluripotency of mESCs in the serum-free condition. Ongoing refinements to components of culture conditions will help to enhance the maintaining of pluripotency and shed more light on pluripotency regulation, more importantly, on how this property can be further exploited for both basic research and regenerative medicine.

## Materials and Methods

### Animal use and care

The specific pathogen-free grade mice (SPF) grade mice, including ICR, C57BL/6n, DBA/2, 129S1/SvlmJ, BDF1, SCID and Oct4-ΔPE-EGFP (OG2) transgenic mice were housed in the animal facility at Tongji University, Shanghai, China. The BDF1 hybrid mice (8–10 weeks old) were obtained by mating male DBA/2 mice with female C57BL/6n mice. All the mice had free access to water and food. All experiments were approved by the Biological Research Ethics Committee of Tongji University and performed following the University of Health Guide for the Care and Use of Laboratory Animals.

### Establishment of mESC

Mouse embryonic stem cells were generated as previously described (Zhang et al., 2020). The female 129S1/SvlmJ mice (6–8 weeks) were intraperitoneally injected with PMSG (5–6 IU, S160106, San-Sheng Pharmaceutical Co.Ltd) and hCG (6–7 IU, B151104, San-Sheng Pharmaceutical Co.Ltd), and then mated with male Oct4-ΔPE-EGFP (OG2) transgenic C57BL/6n mice. Then we collected the 2-cell embryos from the oviducts of the mated female mice at 1.5 days post coitum (d.p.c.) and cultured them in G1 PLUS medium (10136, Vitrolife) until the blastocyst stage. The OG2-positive blastocysts were then randomly individually plated in each well of 96-well plates coated with feeders (mitomycin C-treated MEFs) and were cultured for the expansion of outgrowth. After 6–8 days, the cells were dissociated using 0.25% Trypsin-EDTA (TE, 25200056, Thermo Fisher Scientific) and passaged into 48-well plates (p1, passage 1), followed by a second passaging into 24-well plates (p2) and another third passaging into 6-well plates (p3). The established mESC lines at p3 were genotyped to determine sex. Used primers are listed in Data S5. All the embryos and cells were cultured at 37 °C with 5% CO_2_.

### Culture of mESC

S-mESCs were established and maintained on feeders in canonical serum-containing medium (S medium) including knockout DMEM (10829, Gibco) with 15% fetal bovine serum (FBS, 16000044, Gibco), 1 mmol/L L-glutamine (25030164, Thermo Fisher Scientific), 100× nucleosides (M6250, Sigma-Aldrich), 100× NEAA (TMS-001, Millipore), 0.11 mmol/L 2-mercaptoethanol, 10^3^ U/mL LIF (ESG1107, Millipore) and 100× penicillin/streptomycin (15140122, Gibco). S/2i-mESCs were converted from S-mESCs and further cultured over 15 days on 0.3% gelatin-coated plates in S medium supplemented with 1 μmol/L PD0325901 (S1036, Selleck) and 3 μmol/L CHIR9902 (S1263, Selleck). N/2i-mESCs were converted from S-mESCs and further cultured over 15 days on 0.3% gelatin-coated plates in serum-free chemically defined medium (N medium) supplemented with 1 μmol/L PD0325901 and 3 μmol/L CHIR9902, termed as N/2i medium. N medium includes DMEM/F12 (11320033, Gibco) and Neurobasal (21103049, Gibco) (1:1), 1% N2 (17502048, Gibco), 2% B27 (17504044, Gibco), 1 mmol/L L-glutamine, 0.11 mmol/L 2-mercaptoethanol, 1000 U/mL LIF, 100× penicillin/streptomycin. N/2i + BMP4-mESCs were cultured on 0.3 % gelatin-coated plates in N/2i medium supplemented with 10 ng/mL BMP4 (315-27, Peprotech). N/a2i-mESCs were cultured on 0.3% gelatin-coated plates in N medium supplemented with 1.5 μmol/L CGP77675 (SML0314, Sigma), 3 μmol/L CHIR9902. N/t2i-mESCs were cultured on 0.3% gelatin-coated plates in N medium supplemented with 0.2 μmol/L PD0325901, 3 μmol/L CHIR9902.

### Generation of mCherry-labeled mESCs

The coding sequence of mCherry was cloned and inserted into the FUGW vector to generate the FUGW-mCherry vector. FUGW-mCherry vector was extracted and purified using an EndoFree Plasmid kit (CoWin Biotech Co., Beijing, China). For the generation of lentivirus, 293T cells were transfected with individual vectors combined with psPAX2 and pMD2.G packaging plasmids (5:3:2) using Vigofect (Vigorous Biotechnology Beijing Co., Ltd.). The supernatants containing the virus were collected after 48 h, filtered through a 0.45 mm filter (Merck Millipore), and concentrated by PEG-8000 according to the standard protocols. After concentration, the viruses were resuspended in the corresponding medium, and 2 × 10^4^ mESCs were infected. After 8–10 h viral infection, the cells were washed with PBS and cultured using fresh media. PCR was used to detect exogenous gene integration. mCherry-labeled mESCs could be purified by MoFlo XDP cell sorter (Beckman Coulter).

### Generation of *Ube2s* and *Chmp4b* knockdown or overexpressed mESCs and *Zbtb7a* overexpressed mESCs

The sequence encoding *Ube2s* and *Chmp4b* shRNA was constructed into the vector PLVshRNA (2A)-Puro (VL3104, Inovogen Tech.Co.) and PLKO-Tet-On shRNA (Novartis) for consistent knockdown and inducible knockdown, respectively. The sequence encoding *Ube2s*, *Chmp4b* and *Zbtb7a* was constructed into the vector lenti-EF1α (gift from Bing Zhu Lab, IBP, China). Then the constructed vectors and the scramble vector were separately introduced into mESCs through lentivirus infection as described above. The infected mESCs were further purified by FACS or puromycin screen. PCR was used to detect exogenous gene integration. And the gene expression levels were also detected. A list of the primers used is provided in Data S5. For the inducible knockdown, expression of *Ube2s* and *Chmp4b* were validated after Dox (1 μg/mL) induction. The *Ube2s* and *Chmp4b* shRNA sequences are listed in Data S5.

### Teratoma assay

mESCs were trypsinized for single cell suspension in 250 µL of PBS and 1 × 10^6^ cells were injected subcutaneously into the groin of a female SCID mouse. Four weeks post-injection, tumors were dissected and processed for hematoxylin-eosin (HE) staining by Servicebio Co.Ltd.. Finally, the tumor sections were blindly scored for the presence of each germ layer.

### Chimera assay and fluorescence-activated cell sorting (FACS) based analysis

mCherry labeled Oct4-EGFP+ mESCs were firstly trypsinized and resuspended in FACS buffer (PBS with 2% FBS) to sort mCherry- and EGFP-double positive population by MoFlo XDP cell sorter (Beckman Coulter). Mouse chimera assay was performed as previously described (Chen et al., 2015b). The ICR blastocysts were collected as described above (Establishment of mESC). 10–15 double-positive mESCs were microinjected into a blastocyst using a piezo-actuated microinjection pipette. The manipulated embryos were then transplanted into the uteruses of 2.5 d.p.c. pseudo-pregnant ICR mice. Next, pregnant female ICR mice at 13.5 d.p.c were sacrificed and the uteri were dissected. The chimeric embryos were carefully freed from the myometrium using properly sharpened forceps (3110, Sigma) and imaged with the same exposure intensity by Olympus SZX16 stereo zoom microscope. Besides, gonads were carefully isolated and imaged for EGFP and mCherry signaling. Then tissues from the hindneck and gonad were cut up and further dissociated for analyzing the percentage of EGFP+ and mCherry+ cells by CytoFLEX S (Beckman Coulter). To eliminate the interference of supporting cells and get the authentic germline chimerism, we used the calculated ratio for germline comparison. The germline chimerism = “the percentage of Oct4-EGFP+ cells in E13.5 chimeric gonad” / “mean of the percentage of E13.5 OG2 gonads (*n* = 11, mean = 20.258%)”.

### Tetraploid embryo complementation (TEC) assay

The TEC assay was performed as previously described (Chen et al., 2015b). In short, to produce tetraploid embryos, the late 2-cell stage embryos were first electro-fused and then cultured until 8-cell or morula stage. Then 10–15 individual mESCs were sandwiched between two tetraploid embryos (8-cell or morula stage). After 24 h of culture in G1 PLUS medium, the reconstructed blastocysts were transplanted into the uterus of 2.5 d.p.c. pseudopregnant ICR mice. We carried out the cesarean section at 19.5 d.p.c. and used lactating ICR mice to foster the pups. Meanwhile, some of the recipient pseudopregnant mice were able to deliver full-term pups by themselves. We termed pups “full-term”, “breathing”, and “adult” when they reached the full-term (E19.5), established autonomous respiration after birth, and survived over 5 weeks, respectively. Simple sequence polymorphism (SSLP) analysis was performed (detection site: D2Mit102, D8Mit94 and D11Mit236) to confirm these mice were truly TEC mice. Then adult TEC mice were further mated with ICR mice for the examination of germline transmission competence. The PCR primers for SSLP analysis are listed in Data S5.

### Reverse transcription and reverse transcription quantitative-PCR (RT-qPCR)

For RT-qPCR analysis of indicated mESCs in this study, total RNA was purified using Trizol reagent (Takara) and reverse-transcribed using 5× All-In-One RT Master Mix (G492, ABM) according to the manufacturer’s recommendations. RT-qPCR was performed using SYBR Premix Ex Taq II (RR820B, Takara) and signals were detected with ABI7500 Real-Time PCR System (Applied Bio Systems). The cDNA was diluted 1:10 in nuclease-free ddH_2_O and then used in RT-qPCR analysis. Hypoxanthine-phosphoribosyl-transferase gene (*Hprt*) was used as an endogenous control. Primers are listed in Data S5, and all the primers were synthesized at Genewiz Co. Ltd..

### Immunofluorescent staining

mESCs cultured on cover slides (Biobest) were fixed in 4% paraformaldehyde (PFA) for 30 min and then permeabilized for 15 min with 0.3% Triton X-100 (T8532, Sigma). The slides were blocked with 2.5% bovine serum albumin (BSA) (Sigma) for 1 h at room temperature and incubated with primary antibodies against γH2A.X (9718S, Cell Signaling Technology) overnight at 4 °C. The cells were then washed three times with PBS and incubated with secondary antibodies conjugated to Alexa Fluor 488 for 1 h at room temperature. The nuclei were stained with 4,6-diamidino-2-phenylindole (DAPI) for 15 min at room temperature. The glass coverslips in the glass slide were observed with a ZEISS LSM880 confocal microscope. The captured images were processed and quantified with ZEISS Zen blue edition.

### EdU and 5-EU incorporation assay

For EdU incorporation assay, cells cultured in different conditions were independently treated with EdU (10 μmol/L) for 40 min. Then cells were harvested for fixation, permeabilization, and the Click-iT reaction using Azide 647. Hoechst was used for DNA count staining following the manufacturer’s instructions (BeyoClick™ EdU Cell Proliferation Kit with Alexa Fluor 647, C0081S, Beyotime). Next, cells were analyzed using a Beckman Coulter CytoFLEX S flow cytometer. For 5-EU incorporation assay, cells were treated with 5-EU (1 mmol/L) for 1 h. After fixation and permeabilization, the cells were stained for Alexa Fluor 647 azide (A10277, Invitrogen) and DAPI following the manufacturer’s instructions (Click-iT RNA Imaging Kits, C10329, Invitrogen) and analyzed by ZEISS LSM880 confocal microscope. The captured images were processed and quantified with ZEISS Zen blue edition.

### Karyotyping and G-band karyotype analysis

The karyotype analysis had been described in the previous report (Zhang et al., 2020). Briefly, mESCs were cultured in a corresponding medium with 0.25 μg/mL colcemid (Invitrogen, Thermo Fisher Scientific) for 2.5 h and dissociated and collected with 0.05% Trypsin-EDTA. Then we incubate the cells in a hypotonic solution containing 0.4% potassium chloride and 0.4% sodium citrate at 37 °C for 5 min. After fixed with a methanol/acetic acid mixture (3:1, *v*/*v*), the cells were mounted on coverslips waiting to dry, and then stained with Giemsa at 37 °C for 15 min. The chromosome number of the separated nucleus was counted under an inverted microscope (Leica). For each sample, at least 20 nuclei were analyzed. G-band karyotype analysis of the mESCs was performed at KingMed Diagnostics Group Co., Ltd.

### Western blot

1 × 10^6^ cells were harvest and resuspended in 40 μL 2× protein loading buffer supplemented with 5% β-mercaptoethanol (Amersham, CT) and 40 μL 2× proteinase inhibitor (PI) (k1007A, pexBio). Then the suspensions were boiled at 99 °C for 10 min to denature protein. Prepared proteins were separated by 10% vertical SEMS-polyacrylamide gel (PG112, EpiZyme) based on the different molecular size. The separated proteins were then transferred to a PVDF membrane at 200 mA, 2 h. Next, the membrane was blocked with protein free rapid blocking buffer (PS108, EpiZyme) for 1 h at room temperature and then incubated with primary antibodies overnight at 4 °C on a shaker and with second antibodies at room temperature for 1 h. Last, the protein signals were measured using SuperSignal West Pico PLUS (34580, Thermo Scientific) and visualized with a ChemiDoc MP Imaging System (BioRad). Primary antibodies were used as follows: anti-TUBLIN (T3559, Sigma), anti-UBE2S (14115-1-AP, Proteintech), anti-CHMP4B (13683-1-AP, Proteintech), anti-H2A.X (ab11175, abcam), anti-γH2A.X (9718S, Cell Signaling Technology), anti-ERK1/2 (AF1051, Beyotime), anti-p-ERK1/2 (AF5818, Beyotime).

### Enzyme-linked immunosorbent assay (ELISA)

We performed ELISA following the manufacturer’s instructions (ERK1/2 (pT202/Y204 + Total) ELISA Kit, ab176660, Abcam). Each group has three experiment replicates, and each replicate was performed using 25,000 cells. Specifically, buffers including 1× Cell Extraction Buffer PTR, 1× Wash Buffer PT, 5× Cell Extraction/Enhancer Buffer PTR and Antibody Cocktail needs to be prepared prior to assay. Cells were trypsinized and centrifuged, then the cell pellet was resuspended in 50 μL 1× Cell Extraction Buffer PTR and transferred to individual wells of a plate. The plate was then incubated for 1 h at room temperature on a plate shaker. Each well was washed by 350 µL 1× Wash Buffer PT for 3 times. Then 100 µL of TMB Substrate was added to each well and incubated for 15 min in the dark on a plate shaker. Finally, 100 µL Stop Solution was added to each well. The plate was shaking for 1 min and the OD was recorded at 450 nm.

### Chromatin immunoprecipitation-qPCR (ChIP-qPCR)

ChIP-qPCR were performed as previously described (Le et al., 2021). 1 × 10^4^ cells were harvested per reaction and resuspended in nuclear extraction buffer. Chromatin was fragmented for 8 min using MNase at 25 °C, and stopped in 100 mmol/L EDTA solution, then diluted in ChIP immunoprecipitation buffer (20 mmol/L Tris-HCl pH 8.0, 2 mmol/L EDTA, 15 mmol/L NaCl, 0.1% Triton X-100, 0.1% deoxycholate, 1× EDTA-free protease inhibitor cocktail and 1 mmol/L phenylmethanesulfonyl fluoride (Sigma)). Fragmented chromatin was incubated with 1 μg of antibody–bead complexes (10 μL of protein G Dynabeads, Life Technologies) overnight at 4 °C. IPed complexes were washed twice with 200 μL of low salt wash buffer (20 mmol/L Tris-HCl, pH 8.0, 0.1% SDS, 1% Triton X-100, 0.1% deoxycholate, 2 mmol/L EDTA and 150 mmol/L NaCl) and twice with 200 μL of high salt wash buffer (20 mmol/L Tris-HCl pH 8.0, 0.1% SDS, 1% Triton X-100, 0.1% deoxycholate, 2 mmol/L EDTA and 500 mmol/L NaCl). Protein–DNA complexes were eluted in 100 μL of ChIP hot elution buffer (100 mmol/L NaHCO3 and 1% SDS) for 2 h at 65 °C. IPed material was purified by phenol chloroform, ethanol-precipitated. We diluted the purified DNA 5 times for ChIP-qPCR reaction. Primary antibodies were used as follows: IgG (ABIN101961, Antibodies-Online), H3K4me3 (C15410037, Diagenode), H3K9me3 (39161, Active Motif), H3K27me3(C15410195, Diagenode), H3K36me3(ab9050, Abcam), H3K9ac (zb4441, Abcam), H3K27ac (39133, Active Motif). Primers for ChIP-qPCR were listed in Data S5.

### RNA-Seq

RNA-seq libraries were performed as previously described (He et al., 2018). 200 ng of total RNA calculated by Qubit 2.0 (Invitrogen) was prepared for library construction. RNA-seq libraries were generated using the KAPA Stranded mRNA-Seq kit according to the manufacturer’s manual. In Short, the mRNA was enriched with oligo magnetic beads. Then cDNA was synthesized using random hexamer primers and purified with 1.0× Agencourt AMPure XP beads (Beckman). Finally, the cDNA fragments (approximately 300 bp) linked with sequencing primers were isolated by gel electrophoresis and amplified by PCR. The sequencing was performed by Berry Genomics Co Ltd. using the NovaSeq system developed by Illumina. 2 or 3 biological replicates were analyzed for each treatment condition.

### Whole-genome Bisulfite Sequencing (WGBS)

WGBS libraries had been described previously (Wang et al., 2018). Genomic DNA was isolated using the Wizard Genomic DNA purification kit. 30 ng of DNA calculated by Qubit was used per reaction. The libraries for sequencing were generated with the Pico Methyl-Seq Library Prep Kit following the manufacturer’s manual. Paired-end 150 bp sequencing was performed on a NovaSeq platform by Berry Genomics Co Ltd..

### RNA-seq data analysis

Adapter and low-quality reads were trimmed using cutadapt (v1.18) with parameters: -a AGATCGGAAGAGC -A AGATCGGAAGAGC --trim-n -m 50 -q 20,20. reads were aligned to the mouse reference genome (mm10) using hisat2 (v2.1.0) and sorted with Samtools (v1.9). IGV files were generated using bamCoverage from the deeptools suite (v3.1.3) with parameters: --normalizeUsing RPKM--binSize 25. Gene quantification was performed using feature Counts (v1.6.1) with parameters: -p -C -B. The gene counts were normalized using variance stabilizing transformations methods by DEseq2 and subsequently used for Principal component analysis (PCA) and detection of differentially expressed genes (DEGs). Genes with *P*-values < 0.01 and fold change > 2 were considered as significant DEGs. Gene expression levels (FPKM) were calculated using StringTie and R package ballgown with default parameters. 

### Functional enrichment analysis

Gene ontology analyses were performed using two online platforms, GENEONTOLOGY (96-98) and g: Profiler (Raudvere et al., 2019). For each enrichment analysis, we used Benjamini-Hochberg FDR to calculate the Significance threshold. The data sources we used are GO molecular function, GO biological process, KEGG pathway, and TRANSFAC motifs. Enrichment terms for each functional cluster were summarized to a representative term, and *P*-values or fold change was used to plot. For GSEA analysis, *P*-values were calculated based on one million permutations. For both types of analysis, pathways were considered significant if the FDR-corrected *P*-value was ≤ 0.01.

### WGBS data analysis

Adapter and low-quality sequences were removed by cutadapt (v1.18) as described above. Reads were mapped to a combined genome with mm 10 and 48052 lambda sequence with bsmap (100). The methylation level of each CpG site was estimated using mcall. The methylation level of the same CpG site from multiple replicates was determined using total methylated reads count across replicates versus total reads count across replicates, and CpG sites with less than 3 reads were discarded.

### Quantification and statistical analysis

Most statistical analyses were performed with GraphPad Prism (GraphPad Software, Inc.) or R (www.r-project.org/). Other specific statistical method was performed as indicated in the manuscript or figure legends. Statistical significance was calculated using a Two-tailed unpaired Welch’s *t*-test or a Two-tailed unpaired Student’s *t*-test. ns, not significant. *P < 0.05. **P < 0.01, ***P < 0.001. Regression analysis was also performed by GraphPad.

## Supplementary Information

Below is the link to the electronic supplementary material.

Supplementary file1 (XLSX 3114 kb)

Supplementary file2 (XLSX 47 kb)

Supplementary file3 (XLSX 9632 kb)

Supplementary file4 (XLSX 46 kb)

Supplementary file5 (XLSX 11 kb)

Supplementary file6 (PDF 525 kb)
