# Supplementary material for: BMP4 preserves the developmental potential of mESCs through Ube2s- and Chmp4b-mediated chromosomal stability safeguarding
Source: Protein Cell. 2022 Feb 11;13(8):580–601. doi: 10.1007/s13238-021-00896-x (PMC9232672; doi:10.1007/s13238-021-00896-x)
Supplement: Supplementary file 6 — Supplementary file6 (PDF 525 kb) [file 13238_2021_896_MOESM6_ESM.pdf]

Supplementary Materials for

BMP4 preserves the developmental potential of mESCs through *Ube2s*- and *Chmp4b*-mediated chromosomal stability safeguarding

Mingzhu Wang[1,2,*], Kun Zhao[1,2,*], Meng Liu[1,2,3,*], Mengting Wang[1,2], Zhibin Qiao[1,2,4], Shanru Yi[1,2], Yonghua Jiang[5], Xiaochen Kou[1,2], Yanhong Zhao[1,2], Jiqing Yin[1,2], Tianming Li[1,2], Hong Wang[1], Cizhong Jiang[2,3]†, Shaorong Gao[1,2,4]†, Jiayu Chen[1,2]†.

Supplementary Figure legends

Fig. S1. S/2i-mESCs present a better developmental potential compared to N/2i-mESCs, Related to Fig. 1.

(A) Comparison of the cell cycle of mESCs cultured under S, N/2i and S/2i conditions for 15 days as analyzed by EdU assay. Note that N/2i-mESCs presented an abnormal cell cycle. Data were analyzed from three male mESC lines (#S2, #S3 and #S7).

(B) Karyotype analysis and representative images of S-, N/2i- and S/2i-mESCs.

(C) Karyotype analysis of mESCs (#S7) cultured under S, N/2i and S/2i conditions for 15 and 30 days. Data at day 30 were used to assess the significance. n=2 biological replicates.

(D) Schematic diagram of FACS based chimeric potency assay for accurately determining the *in vivo* differentiation potential of mESCs. Samples include skin, gonads, and other organs of the E13.5 chimeric embryos can be detected.

(E) Representative flow cytometry images indicate the proportion of mCherry+ cells in skin and Oct4-EGFP+ cells in gonad of E13.5 chimeric embryos.

(F) Schematic diagram showing the procedure of classical chimera assay and the time required.

(G) Left, representative images of the gonad (blue dotted line) and mesonephros (red dotted line) of a male and a female E13.5 homozygous OG2-transgenetic mouse embryo. Scale bar, 2 mm. Right, histogram showing the percentage of Oct4-EGFP+ cells in the gonad of E13.5 OG2-transgenetic embryos. Data are shown as mean ±

SEM and analyzed by 11 embryos.

(H) Resorption statistics of S-, N/2i- and S/2i-mESCs in chimera assay. Note that all N/2i-mESCs showed an obviously high resorption rate in chimera assay. Data were calculated from three male mESC lines (#S2, #S3 and #S7).

(I) Confirmation of the indicated all-ESC mice by simple sequence length polymorphism (SSLP) assay. Genomic DNA of C57 and 129 mice were used as positive controls and genomic DNA of DBA/2 mice was used as a negative control.

Data are represented as the mean ± SEM in (A), (G) and (H), the mean ± SD in (C). Statistical analysis was performed using a two-tailed unpaired Welch's t-test. *p < 0.05; **p < 0.01; *** p < 0.001.

Fig. S2. Inactivated TGFβ, BMP, AKT signaling pathways are improved in S/2i condition, Related to Fig. 2.

(A) Volcano Plot showing the global differentially expressed genes (DEGs) between female N/2i- and S/2i-mESCs. Vertical line indicates expression fold change=2, and horizontal line indicates p-value = 0.01.

(B) Heatmap showing the expression pattern of stem cell population maintenance genes (171) in N/2i-mESCs was consistently different from that in S- and S/2i-mESCs. Two male cell lines of each condition were used in this test. Each column represents the means of 2-3 biological replicates. The gene-expression intensity was scaled across samples (red for high expression and blue for low expression). Gene lists are cited from Mouse Genome Informatics (MGI), GO:0019827.

(C) Representative house-keeping genes dysregulated in N/2i-mESCs. Dotted line indicates fold change=±2.

(D) 5-EU incorporation assay indicated an obvious loss of nascent RNA synthesis in N/2i-mESCs compared with S- and S/2i-mESCs. Representative immunofluorescence images of indicated mESCs are shown in the left. Scale bar, 20μm. More than 40 cells were counted for each group. Data are represented as the mean ± SEM. Statistical analysis was performed using a two-tailed unpaired Welch's t-test. *** p < 0.001.

(E) GSEA showing the global changes of EGF receptor signaling pathway (n=104), FGF receptor signaling pathway (n=78), HGF receptor signaling pathway (n=13) and Activin receptor signaling pathway (n=45) in S/2i-mESCs compared to N/2i-mESCs. These gene sets were generated by Gene Ontology Browser on MGI website (http://www.informatics.jax.org/vocab/gene_ontology). NES, normalized enrichment score; FDR, false discovery rate.

(F) Western blot of SMAD5 and p-SMAD5 in mESC line #S2 under indicated culture conditions. The result showed that BMP4 and serum could promote the phosphorylation level of SMAD5.

(G) Genome browser view of Smad1/5 binding sites (shaded in grey) at indicated genome loci of representative Klf family members. ChIP-seq data of Smad1/5 in serum/LIF (Serum) and N2B27/LIF+BMP4 (BMP4) mESCs are cited from Morikawa (Morikawa et al., 2016).

Fig. S3. BMP4 safeguards chromosomal integrity and preserves the developmental potential of mESCs, Related to Fig. 3.

(A) Karyotype analysis of mESCs (line #S7) cultured in N/2i and N/2i+BMP4 conditions with or without feeder for 15 and 30 days. Data at day 30 was used to assess the significance. n=2 biological replicates.

(B) Representative G-banding karyotyping of male mESC line #S2 and #S3 under N/2i+BMP4 condition at day 30.

(C) The level of H2A.X phosphorylation (γH2A.X) was higher in N/2i-mESCs compared to N/2i+BMP4-mESCs. Represent images of mESC line #S2 are shown in the right. Scale bar, 20 μm.

(D) Representative morphological images of mESCs cultured under indicated conditions. The pictures were taken at 54 hours after propagation of 10^5 cells. Scale bar,100 μm.

(E) Cell cycle comparison of mESCs cultured under indicated condition as analyzed by EdU assay. Note that BMP4 supplemented in N/2i condition could maintain a regular cell cycle similar to that in serum conditon. Three male mESC lines (#S2,

#S3 and #S7) were used in this test.

(F) Violin plot showing cell cycle duration of mESCs cultured under indicated conditions. Three male mESC lines (#S2, #S3 and #S7) were used in this test.

(G) Resorption statistics of N/2i- and N/2i+BMP4-mESCs in chimera assay. Note that BMP4 addition greatly reduced the high resorption rate noticed in N/2i-mESCs. Data were calculated from three male mESC lines (#S2, #S3 and #S7).

Data are represented as the mean ± SEM in (A), (C), (E)-(G). Statistical analysis was performed using a two-tailed unpaired Welch's t-test in (A), (C), (F), (G) and a two-tailed unpaired Student's *t*-test in (E). *$p < 0.05$; **$p < 0.01$; *** $p < 0.001$. n.s., not significant.

Fig. S4. *Ube2s* and *Chmp4b* are essential for BMP4-mediated chromosomal integrity safeguarding and pluripotency maintenance, Related to Fig. 4.

(A) RT-qPCR analysis of indicated cell cycle related genes in S-, S/2i-, N/2i- and N/2i+Bmp4-mESCs. *Hprt* was set as an endogenous control. Three male mESC lines (#S2, #S3, and #S7) were tested with 3 biological replicates.

(B) Genome browser view of Zbtb7a binding sites (shaded in orange) at *Ube2s* and *Chmp4b* genome loci. ChIP-seq data of Zbtb7a in N/2i-mESCs was cited from GSE146653 (Yu et al., 2020).

(C) Validation of indicated gene expressions in control and *Zbtb7a*-overexpressed (OE-*Zbtb7a*) mESCs under N/2i condition. A male mESC line #S2 was used in this test. *Hprt* was set as the endogenous control. $n = 3$ replicates. Data are represented as the mean ± SEM. Statistical analysis was performed using a two-tailed unpaired Welch's t-test. *$p < 0.05$; *** $p < 0.001$.

(D) ChIP-qPCR analysis of indicated histone modifications occupancy at promoter regions of *Ube2s* and *Chmp4b* in N/2i- and N/2i+BMP4-mESCs. Two and one potential regulator regions of *Ube2s* and *Chmp4b* were detected, respectively. Relative enrichment was normalized to IgG ChIP signals at the same regions. A male mESC line #S2 was tested with 2 independent experiments. Data are represented as the mean ± SEM. Statistical analysis was performed using a two-

tailed unpaired Welch's t-test. *$p < 0.05$.

Fig. S5. *Ube2s* and *Chmp4b* are essential for BMP4-mediated chromosomal integrity safeguarding and pluripotency maintenance, Related to Fig. 4.

(A) Validation of knockdown efficiency in sh*Ube2s*- and sh*Chmp4b*-mESCs based on consistent (upper) and inducible gene knockdown (lower) system. shRNA-mESCs were generated from line #S2 and cultured in N/2i+BMP4 condition. The scramble shRNA transfected mESCs were set as the control. As for the inducible system, Dox induction was also conducted in control mESCs. *Hprt* was set as an endogenous control. n = 3 replicates.

(B) Representative images of control-, sh*Chmp4b*- and sh*Ube2s*-mESCs after propagation under N/2i+BMP4 condition. The sh*Ube2s*-mESCs showed obvious smaller colonies as compared to control- and sh*Chmp4b*-mESCs. The pictures were taken at 54 hours after propagation of 10^5 cells. Scale bar,100 μm.

(C) Comparison of cell cycle among indicated mESCs as analyzed by EdU assay. Knockdown of *Ube2s* caused a prolongation of the G1 and G2/M phases. n = 3 replicates.

(D) Validation of cell cycle duration in control-, sh*Chmp4b*- and sh*Ube2s*-mESCs. n = 3 replicates.

(E) Western blot showing an increased γH2A.X level in N/2i+BMP4-mESCs after knockdown of *Ube2s* or *Chmp4b* in mESCs.

(F) Representative immunofluorescence images of γH2A.X enrichment in sh*Ube2s*- and sh*Chmp4b*-mESCs related to Fig. 4F. Scale bar, 20 μm.

(G) Western blot validated the decrease of Ube2s and Chmp4b protein levels in mESC line #S2 in inducible gene knockdown system. The scramble ishRNA transfected mESCs after Dox induction was set as the control. KD, knockdown.

(H) Induced knockdown of *Ube2s* or *Chmp4b* in mESC line #S2 for 5 passages caused an increased resorption rate in chimera assay. The control-, ish*Ube2s*- and ish*Chmp4b*-mESCs were cultured under N/2i+BMP4 condition with Dox for 5 passages before microinjection.

(I) Validation of overexpression of *Ube2s* (left) and *Chmp4b* (right) in N/2i-mESCs. mESC line #S2 was used in this test. *Hprt* was set as an endogenous control. n = 3 replicates.

(J) Representative colony morphological images of control-, oe*Ube2s*- and oe*Chmp4b*-mESCs under N/2i condition. The pictures were taken at 54 hours after propagation of 10^5 cells. oe, overexpression. Scale bar,100 μm.

(K) Overexpression of *Ube2s* in N/2i-mESCs could decrease the resorption rate compared to control N/2i-mESCs. oe, overexpression.

Data are represented as the mean ± SEM in (A), (C), (D) and (I). Statistical analysis was performed using a two-tailed unpaired Welch's t-test. *p < 0.05; **p < 0.01; *** p < 0.001. n.s., not significant.

Fig. S6. BMP4 can restore the dysregulated transcriptome and long-termly preserve the pluripotency of N/2i-mESCs, Related to Fig. 5.

(A and B) Representative images of E13.5 chimeric mice generated from mESCs line #S2 cultured under the N/2i+BMP4 (A) and N/2i (B) conditions for 15, 35 and 55 days after transition. Scale bars, 2 mm.

(C) Hierarchical clustering of gene expression profiles based on Pearson correlation coefficient in indicated mESCs cultured under indicated conditions. Colors from blue to red indicate weak to strong correlation.

(D) Heatmap showing the expression level of 4 indicated groups of genes (related to Fig. 2C) in mESCs under different culture conditions. Note that BMP4 could restore the expression of most dysregulated genes in N/2i-mESCs, whereas a2i and t2i did not have a similar role. The gene-expression intensity was scaled across samples (red for high expression and blue for low expression).

Fig. S7. N/2i+BMP4-mESCs exhibit distinct pluripotent features compared with N/a2i- and N/t2i-mESCs, Related to Fig. 6.

(A) Representative morphological images of mESCs cultured under N/2i+BMP4,

N/a2i and N/t2i conditions. mESC line #S2 was used in this test. The pictures were taken at 54 hours after propagation of 10^5 cells. Scale bar,100 μm.

(B) Karyotyping validation of N/2i+BMP4-, N/a2i- and N/t2i-mESCs. mESC line #S2 was used in this test. More than 40 mitosis phases were counted for each group.

(C) Representative images of karyotype in N/2i+BMP4-, N/a2i- and N/t2i-mESCs. Analysis was done in a male mESC line #S2 under indicated culture conditions.

(D) Heatmap showing the expression of representative DNA methylation related genes in indicated mESCs. Two male cell lines of each condition were used in this test. The gene-expression intensity was scaled across samples (red for high expression and blue for low expression).

(E) Volcano plot showing the DEGs between male N/a2i- mESCs versus N/2i-mESCs. Vertical line indicates expression fold change=2, and horizontal line indicates p-value = 0.01.

(F) Volcano plot showing the DEGs between male N/t2i-mESCs versus N/2i-mESCs. Vertical line indicates expression fold change=2, and horizontal line indicates p-value = 0.01.

(G) Heatmap showing the comparation of the representative house-keeping gene expression in indicated mESC lines. The gene-expression intensity was scaled across samples (red for high expression and blue for low expression).

(H) 5-EU incorporation assay in mESC line #S2 under indicated conditions. Representative immunofluorescence images (left) and statistical analysis (right) are shown. The result showed a significant increase of nascent RNA synthesis in N/2i+BMP4-mESCs compared with N/2i-, N/a2i-, and N/t2i-mESCs. More than 40 cells were counted for each group. Scale bar, 20μm.

(I) Karyotyping validation of S-, N/2i- and N/2i-S-mESCs. N/2i-S means that N/2i-mESCs were switched back to S condition for 15 days' culturing. Note that N/2i-S mESCs showed a high proportion of aneuploidy similar to that of N/2i-mESCs. The mESC line #S2 was used in this test. More than 40 mitoses phases were counted for each group. n=3 biological replicates.

Data are represented as the mean ± SEM in (H) and (I). Statistical analysis was

performed using a two-tailed unpaired Welch's t-test. *$p < 0.05$; *** $p < 0.001$, n.s., not significant.

REFERENCES

Morikawa, M., Koinuma, D., Mizutani, A., Kawasaki, N., Holmborn, K., Sundqvist, A., Tsutsumi, S., Watabe, T., Aburatani, H., Heldin, C.H., *et al.* (2016). BMP Sustains Embryonic Stem Cell Self-Renewal through Distinct Functions of Different Kruppel-like Factors. Stem Cell Reports *6*, 64-73.

Yu, S., Zhou, C., Cao, S., He, J., Cai, B., Wu, K., Qin, Y., Huang, X., Xiao, L., Ye, J., *et al.* (2020). BMP4 resets mouse epiblast stem cells to naive pluripotency through ZBTB7A/B-mediated chromatin remodelling. Nat Cell Biol *22*, 651-662.
